# Supplementary material for: Word selectivity in high-level visual cortex and reading skill
Source: Dev Cogn Neurosci. 2018 Sep 29;36:100593. doi: 10.1016/j.dcn.2018.09.003 (PMC6969272; doi:10.1016/j.dcn.2018.09.003)
Supplement: Supplementary file 1 [file mmc1.docx]

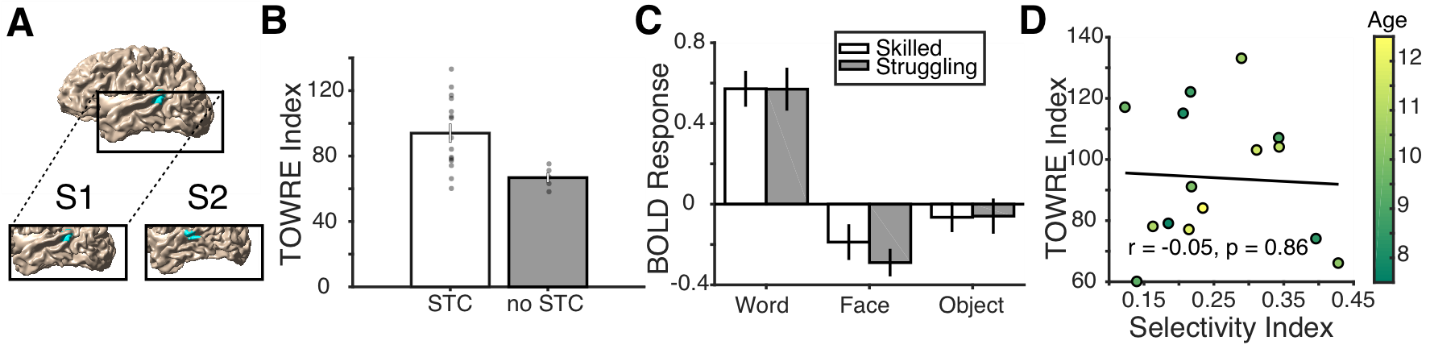


Supplementary Figure 1*. Selectivity in superior temporal cortex (STC) is not correlated with reading skill. (A) STC ROIs defined in individual subjects using a word > face contrast (B) Those with a STC ROI (n = 15) are significantly better readers than those without a STC ROI (n = 5) (*t*(18) = 2.66,* p *= 0.01). (C) Both skilled and struggling readers show high response to words in STC, and no response to other visual stimuli. (D) Word selectivity in STC is not correlated with reading skill.*
